# Satisfaction with Life, Emotions, and Identity Processes in Polish First-Time Mothers and Fathers and Their Child’s Age

**DOI:** 10.3390/ijerph18020799

**Published:** 2021-01-19

**Authors:** Hanna Liberska, Monika Deja

**Affiliations:** Faculty of Psychology, Casimir the Great University, 85-867 Bydgoszcz, Poland; dejam@ukw.edu.pl

**Keywords:** mother, father, identity, satisfaction with life, emotions

## Abstract

The experiences of women regarding conception, the birth of the first child, and care of an infant in the perinatal period have long attracted the attention of researchers, but the knowledge about the experiences of men entering the role of fathers for the first time is still insufficient. The aim of the research was to identify the level of satisfaction with life, emotions and identity formation of first-time parents depending on the gender and age of the child. Seventy-five pairs of Polish first-time parents participated in the study. The research used the SUPIN scale (Polish adaptation of Positive and Negative Affect Schedule), the Satisfaction with Life Scale (SWLS), the Dimension of Identity Development Scale (DIDS), and a questionnaire prepared by the authors. On the basis of the conducted research, it can be concluded that there is a similarity of satisfaction with life, experienced emotions, and identity processes of first-time mothers and fathers, as well as the importance of the child’s age for the specificity of developmental changes in women and men. Understanding development changes which include identity, emotional functioning, and life satisfaction of first-time parents can provide bases for creating supporting programs in the case that problems in undertaking the role of a parent emerge.

## 1. Introduction

Parenthood is a natural consequence of solving the intimacy versus isolation development dilemma which lasts for a period of young adulthood [1]. In the tradition of our cultural field, the aspiration to solve the intimacy versus isolation dilemma, at first, results in entering into marriage and, in more distant consequence, in the birth of the offspring. Although today more and more women and men do not decide to formalize the intimate relationship, reject the ideal family form, and live in different models defined as postmodern or alternative family forms (e.g., LAT–Living Apart Together, DINKS–Double Income No Kids, cohabitations, homosexual and other relationships) [2,3], most of them decide on having a baby.

A favorable factor in making a procreation decision and satisfaction from the fulfilment of the role of mother and father is a structure of the life concept and one’s own person with parenthood fitted into it. As a result of men and women noticing the link between the fulfilment of parenthood and their own psychological development, including identity development, parenthood can become a source of many positive emotions and satisfaction with life [4,5,6,7].

Although the birth of the offspring is a normative event, which is significant for a psychosocial development of a woman and a man, it is also the source of the stress which can disorganize their functioning.

Pregnancy is 12th on the list of stressful life events in terms of stress intensity among adults [8]. Fathering a child is 6th on the modified list of stressful events in non-adults [9]. Both women and men can feel anxious for many reasons. One of them is the sense of threat for autonomy in relations with the partner, in the case of others—fear of losing separate identity or the need of identity change, or the early stage of working on it. Anxiety can be also caused by the belief of the need of day and lifestyle organization change [10]. Anxiety resulting from the threat of losing the position of the most important person in the life of the partner or the spouse can also be a factor delaying or stopping the procreation and becoming a parent. People dependent on external evaluations, whose self-esteem is based on their body advantages/attributes (beauty) or competences and professional status, can delay taking on the maternal or paternal role on account of anxiety associated with the possible loss of physical attractiveness or professional position. Anxiety can be also caused by the current political or economic situation which, in the woman’s or man’s assessment, does not guarantee to provide the child with fair living standards [11]. It follows from the above that at least part of the anxiety and fears associated with the parenthood is settled in social stereotypes and cognitive schemas of the person; however, part of them is settled in the current ecological context [12].

Among the research published so far on the first-time parents, the predominant ones are the explorations involving either women-mothers or men-fathers [13,14], or generations of women [15], or generations of men in the family [16], which reduces chances to recognize resemblances and/or differences associated with sex in examined area. There has been an increasing number of research studies on stress and depression in women and men connected with pregnancy, childbirth, or on stress of the parental role [11,17,18] or stress connected with the need for the change of a lifestyle as a result of the birth of a child [14,16], which introduce new knowledge concerning these important issues. They indicate diversified trajectories of changes in the intensity of positive and negative emotions in women and men [18] and indicators of postpartum depression [14]. Some research points to the significance of the public support for intensifying positive and negative emotions in parents of the small child and to their connections with well-being or with satisfaction with life or satisfaction with marriage relations [18,19,20,21,22].

Psychological knowledge about stress experienced by a woman in connection with getting pregnant, the course of pregnancy and the first childbirth, and post-natal depression is more and more extensive [17,23]; however, the knowledge about events in the life of a man, which are begetting and the birth of his first child and his experiences in the perinatal period, is still insufficient, although more and more research on this problem is beginning to appear [13,14,24].

The concentration of researchers on woman’s experiences partly results from the respect for the importance of the social role of a mother but partly from conviction of multitudes and intensity of stress experienced by women in relation to undertaking the role of a mother. Is coping with the new role of a parent more difficult for women than for men? According to some researchers, the fact that men do not experience a direct contact with a child developing in the womb of a woman can be a factor which limits the time necessary to recognize and manage one’s own feelings and reorganize the identity [25]. However, the process of entering a role of a parent (father and mother) starts much earlier than in the moment of obtaining information about the conception or in the moment of the childbirth, but at the moment when a young person builds the concept of one’s life. Observation of the partner’s changing body, as well as the participation in the childbirth, in a way, opens the doors or “channel” for the man to pass to “be a young father”. The process of a woman entering a role of a mother also starts earlier and is in relation to biological changes during pregnancy. Women can feel very anxious in relation to the course of pregnancy and childbirth, body transformations, partner’s reaction to the birth of a child, the need for the time reorganization, taking care of a small child, and its effects on their employment, economic status, etc. A man expecting a baby can also feel anxiety concerning the health of the partner, course of the childbirth, more distant relations with her after giving birth to child, sexual life, possibility of joining in care of the offspring, and many other issues. In the time of waiting for the birth of a child, in the period of pregnancy, childbirth, and perinatal time, both the man and his partner can experience diversified emotions, both positive and negative, and changes in the level of life satisfaction [18,26], and they also face the challenge of transformation of their identity as a consequence of procreation and increasing the scope of roles and the duties associated with the growing system of the family [16,27]. Some studies on the identity of women and men identify masculinity as a key factor in changing gender inequality in the transition to parenthood [27]. Despite the importance of this issue, there is a clear lack of research on the identity processes of men and women who became parents for the first time [7]. Making an assumption about the interdependence of development changes in the family system constitutes the ground for undertaking the exploration of the problem concerning emotional experiences and identity processes of first-time mother and first-time father in the time of parental role transition [7,28,29].

Performing the role of a parent in a manner approved in a given culture and family tradition requires taking up many new tasks and obligations, including responsibility for raising a child and supporting a partner. It is often associated with the reorganization of activity, resignation from attractive forms of spending time, one’s own ambitions, greater involvement at work, and straining physical and mental strength. This may result in the intensification of negative emotions, lower life satisfaction, and increased work on identity. For many women, the involvement as a first-time mother in new responsibilities leads to greater efficiency in management, new skills and competences, and identity restructuring. The process of adapting to the role of a first-time parent takes place over time.

## 2. Materials and Methods

### 2.1. The Aim of the Research and Research Questions

The presented research was aimed at giving the answer to the question of how emotionality and life satisfaction and dynamics of identity development processes of parents, who in the period of 6 months preceding the examination became fathers and mothers of their first child, were formed. The variables taken into consideration were: identity processes, life satisfaction, intensity of positive and negative emotions, and the age of a child. The results of mothers and fathers were compared.

With reference to the above, the following research questions were created concerning the level of life satisfaction, emotional functioning, and intensity of identity processes of first-time mothers and their partners, i.e., first-time fathers, and their changes in the period covering the first six months after the birth of a child (perinatal period).

1. Do the sex of a parent and the age of a child diversify the level of life satisfaction of first-time parents?
2. Do the sex of a parent and the age of a child diversify the intensity of positive and negative emotions of first-time parents (both permanent, as well as a current, emotional state)?
3. Do the sex of a parent and the age of a child diversify the intensity of identity processes at first-time parents?
4. Are there any relations between life satisfaction of a first-time mother and first-time father and the strength of experienced emotions and identity processes?

### 2.2. Tools

In order to answer research questions, the research included the use of Polish adaptation of the Positive and Negative Affect Schedule (SUPIN scale), the Satisfaction with Life Scale (SWLS), the Dimension of Identity Development Scale (DIDS), and a questionnaire prepared by the authors.

#### 2.2.1. Negative and Positive Emotions

Negative and positive emotions were assessed with the Positive and Negative Affect Schedule (PANAS) [30], in Polish adaptation, of Brzozowski [31]. SUPIN is the Polish adaptation of PANAS. This tool is used for measurement of the intensity of positive and negative emotions. The scale can be used for measurement of the current emotional state and permanent affective states. Two versions of the SUPIN scale were applied: version S30 for measurements of current emotional states and C30 for measurement of relatively enduring dispositions. Each version is composed of a list of adjectives. The person taking part in the study is asked to evaluate on a scale from 1 to 5 the degree (intensity) to which these adjectives describe his/her current state (version S) or his/her enduring dispositions (version C). The results are calculated separately for positive (P subscale) and negative (N subscale) emotions. The points obtained are summed; in each of the scales, it is possible to obtain a results between 15 and 75 points. On the basis of the factor analyses, cluster analyses, and correlations with the results obtained by other tools and intergroup differences, the SUPIN scale could be treated as appropriate. The reliability of the results evaluated by the internal consistency of the scales is high or satisfactory, and the Cronbach α varies from 0.73 to 0.95 for the Polish version of the scale (depending on the version and type of sample). Moreover, version C is characterized by high absolute stability [31].

#### 2.2.2. Satisfaction with Life

This construct was measured using The Satisfaction with Life Scale (SWLS) by Diener, Emmons, Larson, and Griffin [32], in the Polish adaptation of Juczyński [33]. The questionnaire examines the overall assessment of the quality of life. It consists of 5 items, rated on a seven-point scale. The theoretical range of the SWLS scale is 5 to 35. The result of the measurement is the overall level of satisfaction with life. The average test time is 2 min. Regarding reliability, the Cronbach’s alpha reliability SWLS index established in a study of 371 people turned out to be satisfactory at 0.81 for the Polish version of the scale. The scale stability index, determined in the double study of a group of 30 people with an interval of six weeks, was 0.86.

#### 2.2.3. Identity

Identity were assessed with Dimensions of Identity Development Scale (DIDS). The authors of this questionnaire are Luyckx, Schwartz, Berzonsky, Soenens, Vansteenkiste, Smits, and Goossens [34]. It has been adapted to Polish conditions by Brzezińska and Piotrowski [35]. This tool identifies the position of an individual in the five dimensions proposed by Koen Luyckx and colleagues [34], such as: exploration in breadth (EB), exploration in depth (ED), ruminative exploration (ER), commitment making (CM), and identification with commitment (IC). The tool consists of 25 theorems creating 5 scales/dimensions (5 items for each scale). All statements are assessed on a six-point Likert scale, from 1—I completely disagree to 6—I completely agree. For each of the scales, an average result is calculated; the scope of results is located between 1 and 6 points for the scale. The reliability coefficients for the scales (Cronbach’s alpha) of the original version are in the range of 0.79 to 89. The reliability coefficients for the scales (Cronbach’s alpha) of the Polish version are in the range of 0.70 to 0.88.

#### 2.2.4. Demographic Information

The questionnaire proposed by the authors allows to collect basic data, such as age, sex of a parent, place of residence, and age of a child.

### 2.3. Participants

The study involved 75 couples of Polish first-time parents who became parents of their first child 6 months before the study. The age of women ranges from 18 to 37 years (*M* = 26.11; *SD* = 3.99), and the age of men is from 18 to 44 years (*M* = 28.59; *SD* = 4.75). The duration of the couples’ relationship ranges from 0 (they are not in a relationship) to 156 months (*M* = 35.44; *SD* = 27.85).

The data concerning the level of education of examined mothers and fathers are presented in Table 1. In the examined group, the majority were people with secondary education, and the minority were those with primary education.

Taking into consideration the place of residence of the examined pairs, 25 pairs (33.34%) live in the countryside, 13 pairs (17.33%) in a city of up to 25,000 residents, 30 pairs (40.00%) in a city of up to 100,000 residents, 3 pairs (4.00%) in a city of up to 250,000 residents, and 4 pairs (5.33%) in a city of above 250,000 residents.

The parents declared that all children were born on time and do not have any congenital diseases or disorders. The age of the children at the time of the study was from 2 days to 24 weeks (*M* = 14.84; *SD* = 6.19). The children were of both sexes, 40 boys and 35 girls.

### 2.4. Methods of a Statistical Analysis

For checking whether there are important differences between the groups of people examined, the Student’s *t*-test was applied for independent groups. Cohen’s *d* was calculated, which is a quantitative measure of the strength of the given phenomenon, calculated based on collected data (the difference between two averages divided by the standard deviation of data). These statistics are independent of the size of a sample. Pearson’s *r* correlation coefficient was used for the analysis of the relation between the variables.

## 3. Results

### 3.1. Descriptive Statistics

Table 2 presents the descriptive statistics of the variables, i.e. mean value, standard deviation, minimum and maximum result, separately in the group of women and men.

### 3.2. Satisfaction with Life

The first research question concerned the level of life satisfaction of first-time mothers and first-time fathers who became parents for the first time in the period of 6 months before the study. They were also asked about the presence of differences in the level of life satisfaction between first-time mothers and fathers depending on the sex and the age of a child.

The results of statistical analysis of findings concerned life satisfaction of first-time parents are introduced in the Table 3.

The first-time parents who were examined did not differ between themselves in terms of declared life satisfaction (*t* = −0.619; *df* = 148; *p* = 0.537). The results are positioned in 6 sten, which means the average level of life satisfaction (Table 3).

No differences of life satisfaction were detected between mothers whose children were of different ages. Mothers of children up to 3 months of life (*n* = 35) and mothers of children whose age is between 3 to 6 months of life (*n* = 40) had similar life satisfaction (*t* = −0.707; *df* = 73; *p* = 0.482) (Table 4).

The age of children (dichotomized to categories of younger children—from 0 to 3 months of the life—and older—from 3 to 6 months of the life) also did not diversify life satisfaction amongst examined men (*t* = 0.252; *df* = 73; *p* = 0.802) (Table 5).

### 3.3. Negative and Positive Emotions

The second research question concerned the intensity of positive and/or negative emotions related to taking up the role of a first-time mother and father. The question was whether first-time mothers and fathers differ in terms of intensity of positive and negative emotions and whether the age of a child diversifies the level of these emotions.

No significant differences were observed in terms of intensity of positive emotions experienced in the given moment (current) (*t* = −1.005; *df* = 148; *p* = 0.317) and experienced usually (permanent) (*t* = −1.102; *df* = 148; *p* = 0.313) between examined women and men (Table 6). They also did not differ by the level of negative emotions declared at a particular moment (*t* = 1.483; *df* = 148; *p* = 0.140). However, the examined mothers were characterized by significantly higher intensity of negative emotions, being a permanent disposition than fathers (*t* = 2.334; *df* = 148; *p* = 0.021). The effect is moderate (*d* = 0.39) (Table 6). However, it should be noticed that, in first-time mothers, the intensity of positive emotions was high: women’s emotions experienced now (current) have the intensity exceeding the average value (sten 7), and positive emotions experienced by women usually (permanent) are marked by a high intensity (sten between 7 and 8). In first-time fathers, positive emotions experienced now were located in the range of the average result to high (sten between 6 and 7) and were usually being experienced on a high level (sten 8). Both in mothers and in fathers, intensity of negative emotions experienced currently (now) was average (sten 6). Intensity of negative emotions experienced usually by first-time mothers was significantly higher than in first-time fathers (result of women between average and high: sten between 6 and 7).

The age of children does not diversify the emotions experienced by examined first-time mothers. There are no differences in all categories: positive emotions experienced usually (*t* = −1.250; *df* = 73; *p* = 0.215) and in the given moment (*t* = 1.236; *df* = 73; *p* = 0.220) and negative emotions experienced usually (*t* = 0.433; *df* = 73; *p* = 0.667) and in the moment of the examination (*t* = 0.931; *df* = 73; *p* = 0.355) (Table 7).

The age of children does not diversify the emotions experienced by examined first-time fathers, neither positive emotions experienced now (*t* = 0.508; *df* = 73; *p* = 0.613) nor experienced usually (*t* = −1.085; *df* = 73; *p* = 0.282), as well as negative emotions experienced in the given moment (*t* = 0.323; *df* = 73; *p* = 0.747) and negative emotions experienced usually, included as a feature (*t* = −0.061; *df* = 73; *p* = 0.951) (Table 8).

### 3.4. Identity

The third research question concerned the intensity of identity processes in first-time parents and differences in the intensity of identity processes associated with the sex of a parent and the age of a child.

The results of statistical analysis show that the sex of examined parents does not diversify the intensity of identity processes, neither in commitment making (*t* = −1.163; *df* = 148; *p* = 0.247) nor identification with commitment (*t* = −1.032; *df* = 148; *p* = 0.304), exploration in breadth (*t* = 0.066; *df* = 148; *p* = 0.947), exploration in depth (*t* = −0.182; *df* = 148; *p* = 0.856), or ruminative exploration (*t* = −0.647; *df* = 148; *p* = 0.519) (Table 9).

The results of statistical analysis show that the age of children diversifies identity dimensions at examined mothers: mothers of older children are marked by higher intensity of commitment making (*t* = −2.537; *df* = 73; *p* = 0.013) and identification with commitment (*t* = −2.676; *df* = 73; *p* = 0.009), as well as with lower ruminative exploration (*t* = 2.246; *df* = 73; *p* = 0.028). These effects are moderate. Differences are not observed in terms of exploration in breadth (*t* = 1.274; *df* = 73; *p* = 0.207) and exploration in depth (*t* = 0.225; *df* = 73; *p* = 0.823) (Table 10).

The age of children does not diversify the intensity of identity processes for examined fathers in commitment making (*t* = −0.404; *df* = 73; *p* = 0.688), identification with commitment (*t* = 0.047; *df* = 73; *p* = 0.963), exploration in breadth (*t* = 0.328; *df* = 73; *p* = 0.744), exploration in depth (*t* = −0.415; *df* = 73; *p* = 0.679), or ruminative exploration (*t* = 1.232; *df* = 73; *p* = 0.222) (Table 11).

### 3.5. Correlation between Variables

The fourth research question concerned the relation between examined variables: life satisfaction, intensity of positive and negative emotions, and intensity of identity processes at first-time parents.

The results of statistical analysis point to moderate correlations between life satisfaction of examined first-time mothers and their emotionality. The higher the intensity of positive emotions experienced in the given moment (S-P) (*r* = 0.301; *p* < 0.001) and usually (C-P) (*r* = 0.440; *p* < 0.001), the higher life satisfaction of a first-time mother (Table 12). In addition, the lower the level of negative emotions in the given moment (S-N) (*r* = −0.305; *p* < 0.01) and usually (C-N) (*r* = −0.356; *p* < 0.01), the higher life satisfaction of a first-time mother.

In first-time fathers, moderate and positive correlations concern the relation between life satisfaction and intensity of positive emotions in the given moment (S-P) (*r* = 0.431; *p* < 0.001) and usually (C-P) (*r* = 0.459; *p* < 0.001). The relation between life satisfaction and intensity of negative emotions usually (C-N) is weak and negative (*r* = −0.282; *p* < 0.05). No relation was observed between life satisfaction and negative emotions experienced in the given moment (S-N) (*r* = −0.198; *p* > 0.05) (Table 12).

In the group of women, there are moderate positive relations between life satisfaction and commitment making (CM) (*r* = 0.502; *p* < 0.001), identification with commitment (IC) (*r* = 0.431; *p* < 0.001), and moderate negative relation between satisfaction and ruminative exploration (ER) (*r* = −0.502; *p* < 0.001) (Table 13).

In fathers, there is a strong positive correlation between life satisfaction and commitment making (CM) (*r* = 0.575; *p* < 0.001) and identification with commitment (IC) (*r* = 0.629; *p* < 0.001), strong negative correlation of life satisfaction and ruminative exploration (ER) (*r* = −0.537; *p* < 0.001), and weak negative correlation between life satisfaction and exploration in breadth (EB) (*r* = −0.244; *p* < 0.05) (Table 13). The strength of all correlations is higher in the group of first-time fathers than in the group of first-time mothers.

In women, commitment making moderately correlates with all dimensions of examined emotionality—positively with positive emotions experienced now (*r* = 0.415; *p* < 0.001) and usually (*r* = 0.433; *p* < 0.001) and negatively with negative emotions experienced now (*r* = −0.438; *p* < 0.001) and usually (*r* = −0.469; *p* < 0.001). Similarly moderate correlations concern the identification with commitment (IC), which correlates positively with positive emotions experienced now (*r* = 0.438; *p* < 0.001) and usually (*r* = 0.484; *p* < 0.001) and negatively with negative emotions experienced now (*r* = −0.409; *p* < 0.001) and usually (*r* = −0.371; *p* < 0.01). In this group, weak negative correlations were also noted between ruminative exploration (ER) and positive emotions now (*r* = −0.253; *p* < 0.05) and usually (*r* = −0.320; *p* < 0.01) and positive correlation between intensity of ruminative exploration (ER) and negative emotionality included as the permanent disposition (*r* = 0.259; *p* < 0.05). Additionally, in women, there is a weak positive link between the exploration in depth (ED) and intensity of positive emotions experienced currently (*r* = 0.282; *p* < 0.05) (Table 14).

In men, commitment making (CM) correlates weakly positively with emotions experienced in the moment of the examination (*r* = 0.247; *p* < 0.05), moderately positively with positive emotions experienced usually (*r* = 0.353; *p* < 0.01), and weakly and negatively with negative emotions in this moment (*r* = −0.288; *p* < 0.05) and usually (*r* = −0.275; *p* < 0.05). Identification with commitment (IC) is also in the moderate positive connection with intensity of positive emotions now (*r* = 0.334; *p* < 0.01) and usually (*r* = 0.427; *p* < 0.001) and in the moderate negative connection with negative emotions now (*r* = −0.311; *p* < 0.05) and usually (*r* = −0.357; *p* < 0.01). Ruminative exploration (ER) correlates moderately and negatively with positive emotions experienced now (*r* = −0.302; *p* < 0.05) and usually (*r* = −0.425; *p* < 0.001) and weakly positively with negative emotions displayed usually (*r* = 0.289; *p* < 0.05) (Table 14).

## 4. Discussion

The aim of the examination was the analysis on how an emotionalism, satisfaction from the life, and dynamics of identity processes develop in first-time parents. Seventy-five parent couples, who in the period of 6 months preceding the examination became fathers and mothers of their first child, took part in the examination. The analyses showed that mothers and fathers did not differ between themselves in satisfaction from life or intensity of positive and negative emotions, except for negative emotions experienced usually, where the level is higher in mothers. The age of the child does not diversify these variables, either. Moreover, the examined parents do not differ from each other significantly in terms of intensity of identity processes. The age of the children diversifies the dimensions of identity in mothers but not in fathers.

As it was mentioned, life satisfaction of first-time mothers and fathers is similar and has an average level. Age of a child does not diversify the level of life satisfaction of women and men. Life satisfaction of first-time parents is not only influenced by requirements of parental role but also one’s health and economic condition. Support from national institutions and regulations are also significant for life satisfaction of first-time parents. In Poland, such support is ensured and can result in the relative stability of the level of life satisfaction of parents in the first months after the birth of a child. Although the findings of our own studies do not provide the grounds to conclude on any changes to life satisfaction of first-time mothers and fathers in the first half of a year of a child’s life, it is impossible, on the current research stage, to reject the possibility that such changes occur while the child goes through the next development periods. This assumption is empirically confirmed in findings of some research showing that the life satisfaction of parents in a family stage with a small child is higher than in later stages [19,36]. Our studies did not confirm the reports of other researchers that woman’s satisfaction and her feeling of happiness were greater after the childbirth and gradually lessened, unlike changes reported by first-time fathers [19,20]. In the reported examinations, such direction of changes of life satisfaction of first-time mothers and diversifying levels of life satisfaction depending on the sex of a parent and the age of a child were not confirmed. However, the current economic and health condition of society (pandemic) can modify previous conclusions concerning the level of satisfaction and its changes in the family life cycle [10,37,38].

The obtained results show that, in the period of the first six months of the offspring, positive strong emotions outweigh negative emotions of a first-time mother and a first-time father. Their emotions are negative on the average level, except for negative emotions appearing usually at first-time mothers. The strength of positive and negative emotions experienced both usually and now by first-time mothers of children up to 3 months old does not differ significantly from the strength of emotions of mothers whose children are in the second quarter of the first year of life (from 3 to 6 of months old). Similar conclusions also concern first-time fathers. Emotions of first-time parents seem relatively stable through the first half a year of a child’s life. Thus, the period of caring for a small child is marked with positive emotions, the strength of which outweighs the strength of negative emotions experienced by first-time parents. Problems associated with the care and upbringing of a small child that appear in the first months of their life do not lead to reducing the strength of positive emotions, neither by the mother nor the father.

Naturally, it is not possible to exclude that the source of positive emotions is not limited to a baby but is also in other experiences not connected directly with performing the parental role by the woman and the man. A source of strong positive emotions can be a result of sexual life with the partner, an interest of distant relatives and acquaintances, and increasing the circle of acquaintances with other children’s parents. Parenthood also changes the social status of the woman and the man, i.e., “being a parent” can be perceived as a specific “social promotion” because it strengthens their position in the world of adults.

The results of research confirmed the link between experienced emotions and the level of life satisfaction of a first-time mother and the first-time father. The level of life satisfaction of first-time fathers is more strongly connected with positive emotions experienced usually and in the given moment than with negative emotions. An assumption appears that first-time fathers better cope with unpleasant emotions appearing at current situations than first-time mothers. First-time fathers seem to be more emotionally stable than the mothers of their children. Emotional functioning of first-time fathers can have a “protective” or stabilizing effect, or provide balance against emotional experiences of first-time mothers, thus it having positive importance for the psychological condition of women. It is not possible to reject the assumption that the independence of the level of life satisfaction of first-time fathers of temporary negative emotional states is an effect of socialization and creating the image of a strong man in our culture, who does not break down under the influence of unpleasant experiences or everyday problems [39]. A first-time father, who is perceived by the mother of the child (and the child) as a man who is emotionally stable and satisfied from life, constitutes the source of her emotional support in moments of temporary emotional breakdown and depressed mood in relation to problems appearing, among others, in the care of a small child. The support given by the partner is significant for emotions and life satisfaction of the partner receiving it [40].

It occurred that first-time mothers and first-time fathers do not differ in intensity to identity processes in any dimension. The differences of intensity of three dimensions identity associated with the age of a child were recorded in women. First-time mothers of children between 3 and 6 months of life show higher intensity of commitment making processes and the identification with commitment in comparison to mothers of younger children. A different distinction was recorded in the reference to rumination. Intensity of ruminative exploration indicates the level of anxiety and the difficulty in engaging in the areas important for the identity development. Weaker intensity of the rumination processes of mothers of “older children” can be interpreted as the sign of an increase of their competency feeling and self-confidence, which results from higher and higher effectiveness in coping with the motherly role, as well as with other roles performed by a woman (intimate partner, professional, etc.). Higher intensity of identity processes with commitment degree in mothers of “older children” can be accepted as the indicator of pleasure increase in taking up the new motherly role, in which the source can be competence growth perceived by the woman and the abilities associated with the care of the offspring, as well as with other roles and development changes observed in the child.

Higher intensity of commitment making processes by mothers of “older” children than by mothers of “younger” children can be interpreted as the indicator of increasing approval of the procreation decision and its consequence for the further course of life.

The results of this research show the long-lasting duration of the identity reconstruction processes of the woman initiated with making a decision on the conception. The childbirth does not stop woman’s identity changes, but it is only a stage on the way to “being a mother”. The result encourages continuation of the exploration of the transition problem to the identity of the mother and its changes over time.

No differences in intensity of identity processes associated with the age of the child were detected in first-time fathers. It is possible to explain this result with the time shift of entering a parental role by the woman and the man. It is not possible to reject an assumption that transition to the role of a father starts later than transition to the role of a mother, which is stimulated by physiological processes in the body of a pregnant woman [41].

The lack of differences in intensity of identity processes associated with the age of a child of first-time fathers is possible to interpret as the indicator of less involvement of a man into the family identity and stronger involvement in the professional or civil identity [2]. It can result from the preference of a man of the traditional model of father as a “head of the family” or the model of the “absent father” in the family [39]. This assumption is confirmed by the result showing the negative association with life satisfaction and exploration in breadth of men. First-time fathers feeling the need for exploration in different roles, working them or adjusting to the requirements of a new role of a “father” in relation to the birth of a child, may result in lowering life satisfaction. In first-time fathers, similarly to first-time mothers, positive links were noted between life satisfaction and commitment making, identification with commitment and negative links between satisfaction and ruminative exploration. Feelings of coping with the role of a parent results, as noted above, in the intensity of identity processes with the role’s obligations, and it arouses stronger positive emotions and strengthens the belief in the rightness of this commitment, and, consequently, life satisfaction intensifies.

The situation when a first-time parents faces the need to take up a new role—the role of a mother or father of one’s first child—forces the need for transforming the current organization of social roles performed so far [42,43]. Successes in coping with a new parental role are the source of positive emotions, and failures are the source of negative emotions. It can require transforming the structure of identity, with changes of intensity of identity processes, such as exploration in depth, exploration in breadth, identification with commitment, commitment making, and rumination [44]. The restructuring of one’s own identity by the first-time father and mother is a necessary condition for effective coping with the increased or modified repertoire of roles. Psychological knowledge of the subject is very much limited [7,45].

The results of our study did not confirm the diversity of trajectory of intensity changes of positive and negative emotions being connected with the indicators of depression in women and men, which is probably because people participating in the examination did not report any symptoms of the post-natal depression. In this study, the link between satisfaction with life and intensity of emotions experienced by mothers and fathers was confirmed [18,19,20,21,22].

The research referred to in the article concentrated on parents who, for the first time, have experienced pregnancy, childbirth, and care of the baby. According to the authors, it is worth stressing the fact that examinations involved pairs of parents, which enabled comparison of selected aspects of functioning and development of both women and men taking up parental roles for the first time. Based on the results of the research, the resemblance of satisfaction with life and experienced emotions by the first-time father and the first-time mother was confirmed. On the one hand, the resemblance of the structure of the identity of parents was also recognized, and, on the other hand, specific differences in the intensifying of identity dimension was revealed, in the first-time father and mother associated with the age of the child. Recorded differences point at the advantage of the traditional model of the identity in the first-time father in the Polish culture. (However, it needs to be stressed that the examined group was not very numerous.) They are frontier studies, and, on that account, it is not possible to conduct discussion on the obtained results with the extended reference to other psychologists’ research. However, the obtained results match analyses conducted by sociologists on the cultural changes concerning identity of fathers, which points to two attitudes towards fatherhood: traditional (the father as the breadwinner) and modernist (the father as the caretaker and the educator) and to their cultural diversity [27]. The explanation of both reasons and the psychological consequences of separate trajectories of changes in the area of identity of the first-time mothers and the first-time fathers recognized in the current study requires the exploration to be continued since the obtained results seem to show, according to the authors, certain limitation of social-cultural factors of the processes of transition to parenthood. The review of the literature on the subject points to the unique character of the examinations reported in the article, which justifies presenting their results on the international forum.

The presented research is not free from limitations. The level of examined variables, such as satisfaction with life or intensity of positive and negative emotions, is also affected by the other factors: the kind of the relation the parents stay in (marriage or cohabitation), satisfaction with the relationship, and received support from the closest people, as well as the socioeconomic status of the family or the health condition of the parents and the child. In the current stage of the research, it is not possible to reject the assumption that the development of the identity and experienced emotions in first-time parents are also connected with the fact of whether the pregnancy was planned or not, as well as with the time passed from the decision to have a child to the conception or from the problems experienced with the conception and the specialist support (e.g., with the help of in vitro) [46]. It is worth expanding future research to these aspects. It requires increasing the number of people examined. Currently, research is being conducted on the significance of the consistency of pregnancy and practical plans of a woman and a man on the structure of their identity, feeling of effectiveness, and satisfaction with life.

## 5. Conclusions

On the basis of the conducted research, it can be concluded that there is a similarity of satisfaction with life, experienced emotions, and identity processes of first-time mothers and fathers, as well as the importance of the child’s age, for the specificity of developmental changes in women and men. Understanding development changes, which include identity, emotional functioning, and life satisfaction, of a first-time parent can provide bases for creating supporting programs in case the problems in undertaking the role of a parent emerge.

## Figures and Tables

**Table 1:** The educational level of examined people.

*Men* *Women*	Primary	Vocational	Secondary	Higher	Sum
Primary	0 (0%)	1 (1.33%)	0 (0%)	0 (0%)	1 (1.33%)
Vocational	1 (1.33%)	4 (5.34%)	3 (4.00%)	0 (0%)	8 (10.67%)
Secondary	1 (1.33%)	15 (20.00%)	16 (21.34%)	6 (8.00%)	38 (50.67%)
Higher	0 (0%)	3 (4.00%)	7 (9.33%)	18 (24.00%)	28 (37.33%)
**Sum**	2 (2.66%)	23 (30.67%)	26 (34.67%)	24 (32.00%)	75 (100%)

**Table 2:** Descriptive statistics.

	Women		Men	
	*M*(*SD*)	*Min-Max*	*M*(*SD*)	*Min-Max*
**SWLS**	20.73 (6.84)	8.00–32.00	21.37 (5.77)	8.00–33.00
**SUPIN-S-P**	43.42 (10.27)	19.00–67.00	45.16 (10.83)	19.00–70.00
**SUPIN-S-N**	27.19 (14.10)	15.00–75.00	24.09 (11.28)	15.00–75.00
**SUPIN-C-P**	48.74 (10.05)	29.00–74.00	50.38 (9.60)	26.00–75.00
**SUPIN-C-N**	28.74 (11.01)	15.00–62.00	24.86 (9.12)	15.00–59.00
**DIDS-CM**	4.03 (0.99)	1.80–6.00	4.21 (0.86)	2.00–6.00
**DIDS-IC**	4.10 (0.76)	2.00–5.80	4.23 (0.81)	2.00–6.00
**DIDS-EB**	3.54 (0.78)	1.80–5.20	3.53 (0.70)	2.00–5.20
**DIDS-ED**	3.41 (0.76)	1.60–4.80	3.43 (0.67)	1.60–4.80
**DIDS-ER**	2.92 (0.83)	1.40–4.60	3.01 (0.83)	1.40–4.80

**Table 3:** The differences between women and men in terms of life satisfaction.

	Women *M*(*SD*)	Men *M*(*SD*)	*t*(148)	*p*	*d* Cohen
**SWLS**	20.73 (6.84)	21.37 (5.77)	−0.619	0.537	-

**Table 4:** The differences between women in terms of life satisfaction taking into consideration the age of a child.

	Up to 3 m.l. *M*(*SD*)	3–6 m.l. *M*(*SD*)	*t*(73)	*p*	*d* Cohen
**SWLS**	20.12 (7.26)	21.24 (6.53)	−0.707	0.482	-

**Table 5:** The differences between men in terms of life satisfaction taking into consideration the age of a child.

	Up to 3 m.l. *M*(*SD*)	3–6 m.l. *M*(*SD*)	*t*(73)	*p*	*d* Cohen
**SWLS**	21.56 (5.63)	21.22 (5.95)	0.252	0.802	-

**Table 6:** The differences between women and men in terms of intensity of positive and negative emotions.

	Women *M*(*SD*)	Men *M*(*SD*)	*t*(148)	*p*	*d* Cohen
**SUPIN-S-P**	43.42 (10.27)	45.16 (10.83)	−1.005	0.317	-
**SUPIN-S-N**	27.19 (14.10)	24.09 (11.28)	1.483	0.140	-
**SUPIN-C-P**	48.74 (10.05)	50.38 (9.60)	−1.102	0.313	-
**SUPIN-C-N**	28.74 (11.01)	24.86 (9.12)	2.334	0.021	0.39

**Table 7:** The differences between the intensity of positive and negative emotions of first-time mothers taking into consideration the age of children.

	Up to 3 m.l. *M*(*SD*)	3–6 m.l. *M*(*SD*)	*t*(73)	*p*	*d* Cohen
**SUPIN-S-P**	41.82 (11.12)	44.78 (9.42)	−1.236	0.220	-
**SUPIN-S-N**	28.85 (15.09)	25.80 (13.26)	0.931	0.355	-
**SUPIN-C-P**	47.12 (10.82)	50.05 (9.31)	−1.250	0.215	-
**SUPIN-C-N**	29.36 (11.52)	28.24 (10.69)	0.433	0.667	-

**Table 8:** The differences between the intensity of positive and negative emotions at first-time fathers taking into consideration the age of children.

	Up to 3 m.l. *M*(*SD*)	3–6 m.l. *M*(*SD*)	*t*(73)	*p*	*d* Cohen
**SUPIN-S-P**	45.88 (11.45)	44.59 (10.41)	0.508	0.613	-
**SUPIN-S-N**	24.56 (10.60)	23.71 (11.93)	0.323	0.747	-
**SUPIN-C-P**	49.03 (10.98)	51.46 (8.32)	−1.085	0.282	-
**SUPIN-C-N**	24.79 (7.81)	24.93 (10.21)	−0.061	0.951	-

**Table 9:** The differences between first-time parents in terms of intensity of identity processes.

	Women *M*(*SD*)	Men *M*(*SD*)	*t*(148)	*p*	*d* Cohen
**CM**	4.03 (0.99)	4.21 (0.86)	−1.163	0.247	-
**IC**	4.10 (0.76)	4.23 (0.81)	−1.032	0.304	-
**EB**	3.54 (0.78)	3.53 (0.70)	0.066	0.947	-
**ED**	3.41 (0.76)	3.43 (0.67)	−0.182	0.856	-
**ER**	2.92 (0.83)	3.01 (0.83)	−0.647	0.519	-

**Table 10:** The differences in identity among first-time mothers taking into consideration the age of children.

	Up to 3 m.l. *M*(*SD*)	3–6 m.l. *M*(*SD*)	*t*(73)	*p*	*d* Cohen
**CM**	3.72 (1.01)	4.28 (0.91)	−2.537	0.013	0.58
**IC**	3.83 (0.78)	4.30 (0.70)	−2.676	0.009	0.64
**EB**	3.67 (0.77)	3.43 (0.79)	1.274	0.207	-
**ED**	3.43 (0.83)	3.39 (0.70)	0.225	0.823	-
**ER**	3.16 (0.92)	2.73 (0.71)	2.246	0.028	0.53

**Table 11:** The differences in identity among first-time fathers taking into consideration the age of children.

	Up to 3 m.l. *M*(*SD*)	3–6 m.l. *M*(*SD*)	*t*(73)	*p*	*d* Cohen
**CM**	4.16(0.85)	4.24(0.87)	−0.404	0.688	-
**IC**	4.24(0.85)	4.23(0.79)	0.047	0.963	-
**EB**	3.56(0.70)	3.50(0.71)	0.328	0.744	-
**ED**	3.39(0.65)	3.46(0.69)	−0.415	0.679	-
**ER**	3.15(0.83)	2.90(0.83)	1.232	0.222	-

**Table 12:** Pearson’s *r* correlation coefficients between intensity of positive and negative emotions and life satisfaction at examined parents.

	SUPIN-S-P		SUPIN-S-N		SUPIN-C-P		SUPIN-C-N	
	Women	Men	Women	Men	Women	Men	Women	Men
**SWLS**	0.301 **	0.431 ***	−0.305 **	−0.198	0.440 ***	0.459 ***	−0.356 **	−0.282 *

* *p* < 0.05; ** *p* < 0.01; *** *p* < 0.001.

**Table 13:** Pearson’s *r* correlation coefficients between the dimensions of identity and life satisfaction at examined parents.

	CM		IC		EB		ED		ER	
	Women	Men	Women	Men	Women	Men	Women	Men	Women	Men
**SWLS**	0.502 ***	0.575 ***	0.431 ***	0.629 ***	−0.179	−0.244 *	−0.101	−0.227	−0.502 ***	−0.537 ***

* *p* < 0.05; ** *p* < 0.01; *** *p* < 0.001.

**Table 14:** Pearson’s *r* correlation coefficients between the intensity of positive and negative emotions and the dimensions of identity.

	CM		IC		EB		ED		ER	
	Women	Men	Women	Men	Women	Men	Women	Men	Women	Men
**SUPIN-S-P**	0.415 ***	0.247 *	0.438 ***	0.334 **	0.195	0.046	0.282 *	−0.109	−0.253 *	−0.302 *
**SUPIN-S-N**	−0.438 ***	−0.288 *	−0.409 ***	−0.311 *	−0.047	−0.023	−0.022	0.074	0.233	0.167
**SUPIN-C-P**	0.433 ***	0.353 **	0.484 ***	0.427 ***	0.093	0.014	0.134	−0.113	−0.320 **	−0.425 ***
**SUPIN-C-N**	−0.469 ***	−0.275 *	−0.371 **	−0.357 **	−0.023	0.141	−0.060	0.143	0.259 *	0.289 *

* *p* < 0.05; ** *p* < 0.01; *** *p* < 0.001.

## Data Availability

The data presented in this study are available on request from the corresponding author. The data are not publicly available due to privacy.
